# Dispersal shapes compositional and functional diversity in aquatic microbial communities

**DOI:** 10.1128/msystems.01403-24

**Published:** 2024-11-18

**Authors:** Angel Rain-Franco, Alizée Le Moigne, Lucas Serra Moncadas, Marisa O. D. Silva, Adrian-Stefan Andrei, Jakob Pernthaler

**Affiliations:** 1 Limnological Station, University of Zurich, Zurich, Switzerland; 2 Institut National de la Recherche Scientifique (INRS), Centre Eau, Terre et Environnement, Québec, Canada; 3 onCyt Microbiology AG, Zurich, Switzerland; Swansea University, Swansea, United Kingdom

**Keywords:** assembly processes, coexistence, carbon use efficiency, community functioning, Elo-rating, dispersal limitation, homogenizing dispersal

## Abstract

Segregation and mixing shape the structure and functioning of aquatic microbial communities, but their respective roles are challenging to disentangle in field studies. We explored the hypothesis that functional differences and beta diversity among stochastically assembled communities would increase in the absence of dispersal. Contrariwise, we expected biotic selection during homogenizing dispersal to reduce beta and gamma diversity as well as functional variability. This was experimentally addressed by examining the compositional and functional changes of 20 freshwater bacterial assemblages maintained at identical conditions over seven growth cycles for 34 days and subjected to two consecutive dispersal regimes. Initial dispersal limitation generated high beta diversity and led to the repeated emergence of community types that were dominated by particular taxa. Compositional stability and evenness of the community types varied over successive growth cycles, reflecting differences in functional properties. Carbon use efficiency increased during cultivation, with some communities of unique composition outperforming the replicate community types. Homogenizing dispersal led to high compositional similarity and reduced gamma diversity. While a neutral and a competition-based (Elo-rating) model together largely explained community assembly, a pseudomonad disproportionally dominated across communities, possibly due to interaction-related genomic traits. In conclusion, microbial assemblages stochastically generated by dispersal limitation can be gradually “refined” into distinct community types by subsequent deterministic processes. Segregation of communities represented an insurance mechanism for highly productive but competitively weak microbial taxa that were excluded during community coalescence.

**IMPORTANCE:**

We experimentally assessed the compositional and functional responses of freshwater bacterial assemblages exposed to two consecutive dispersal-related events (dispersal limitation and homogenizing dispersal) under identical growth conditions. While segregation led to a decreased local diversity, high beta diversity sustained regional diversity and functional variability. In contrast, homogenizing dispersal reduced the species pool and functional variability of the metacommunity. Our findings highlight the role of dispersal in regulating both diversity and functional variability of aquatic microbial metacommunities, thereby providing crucial insight to predict changes in ecosystem functioning.

## INTRODUCTION

Microbial community assembly involves stochastic processes like passive dispersal, drift, and diversification, leading to random fluctuations in species abundances (1), and deterministic, niche-related phenomena that affect species through abiotic and biotic selection (2). The relative importance of different assembly processes may shift during succession, from predominantly stochastic during initial colonization to increasingly deterministic in mature communities (3–5).

This transition is disrupted if extremely low and high dispersal rates override deterministic abiotic selection (6). For instance, dispersal limitation promoted the emergence of distinct microbial assemblages in identical bioreactor habitats (7) and lake water microcosms (8). Conversely, homogenizing dispersal mitigated the effects of abiotic selection and increased community similarity (9–11). Moreover, dispersal need not be a continuous process but also encompasses extremes like transient isolation or total coalescence (12). While the interplay of dispersal and biotic selection is not fully understood, evidence suggests that antagonistic and facilitative interspecific interactions, modified by historical contingency (13, 14), shape community dynamics upon habitat colonization (6). Moreover, similar climax communities may emerge despite moderate dispersal (15), indicating that biotic interactions may act as stabilizing filters for community structure.

Metacommunities are networks of local communities interconnected by source-sink dynamics (9). The spatial insurance hypothesis predicts that dispersal mitigates the negative effect of suboptimal local (abiotic or biotic) conditions (16). Neighboring communities may rescue depleted ones via dispersal to circumvent local extinction and preserve metacommunity diversity. However, connectivity is a double-edged sword, as the isolation of local communities may protect species from superior competitors (17–19). Such competitively weak rare species may promote functional diversity (20), and thus possibly affect community performance.

We experimentally investigated the interplay between dispersal-related processes and biotic interactions in shaping bacterial community structure and functioning. Dispersal limitation served to generate parallel bacterial assemblages with contrasting composition and functioning at identical environmental conditions (8). We hypothesized (i) that functional differences and beta diversity among stochastically assembled communities would increase during semi-continuous cultivation in the absence of dispersal, but (ii) that subsequent homogenizing dispersal would cause a decrease in beta and gamma diversity, as well as a reduction in functional variability due to biotic selection of disproportionally competitive populations from the metacommunity. Finally, we assessed the implications of species interactions on the observed decrease in beta and gamma diversity, and functional variability, following homogenizing dispersal (i.e., mixing of all communities).

## RESULTS

### Initial colonization and compositional changes during semi-continuous growth cycles

**Fig 1:**
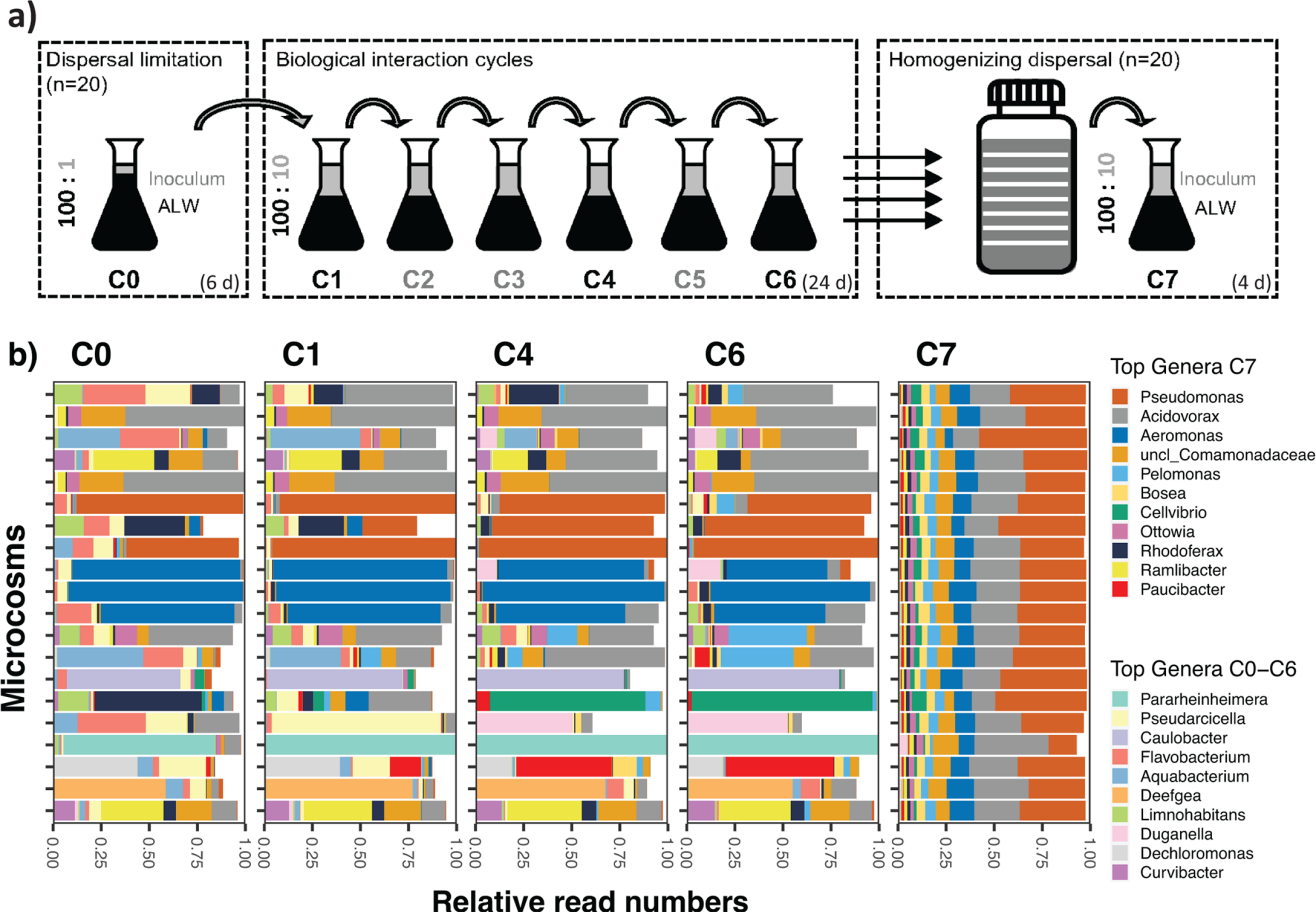
**(a**) Schematic depiction of the experimental design, simulating dispersal limitation (left panel), biological interactions in semi-continuous cultures (center panel), and a homogenizing dispersal event (right panel) over seven consecutive growth cycles (C0–C7). (**b**) Proportions of reads of 16S rRNA genes affiliated with the 10 most abundant genera in metagenomes from the experimental communities at C0, C1, C4, C6, and C7.

We conducted a 34-day culture experiment in artificial lake water using 20 parallel freshwater bacterial assemblages over six semi-continuous growth cycles (Fig. 1a). The putative 16S reads represented 0.04% ± 0.04% (mean ± standard deviation) of total metagenomic reads per sample (Table S1). Altogether, 118 bacterial genera were identified, with *Pseudomonas* (10.9%), *Flavobacterium* (9.2%), *Aeromonas* (6.7%), *Acidovorax* (5.9%), and *Limnohabitans* (5.9%) being the most abundant ones (Table S2).

Experimentally induced dispersal limitation (growth cycle 0, C0) resulted in communities with distinct structures (Fig. 1). *Acidovorax*, *Aeromonas,* and *Pseudomonas* were both, abundant (>30% of reads) and prevalent (present in 20, 15, and 15 microcosms, respectively). C0 was also characterized by genera that were only abundant in single microcosms, such as *Rhodoferax*, *Caulobacter*, *Rheinheimera,* and *Deefgea* (Fig. 1b).

Initially prevalent taxa like *Acidovorax* and *Pseudomonas* persisted from the growth cycle 1 to 6 (C1–C6). Some rarer taxa, such as *Pelomonas*, *Cellvibrio*, *Paucibacter*, and *Duganella*, also increased in abundance over the growth cycles but remained restricted to few microcosms (Fig. 1b). In C6, half of the communities were dominated by *Acidovorax*, *Pseudomonas,* or *Aeromonas* (i.e., >40% of total read numbers), while the other half were unique with respect to the most abundant taxon.

**Fig 2:**
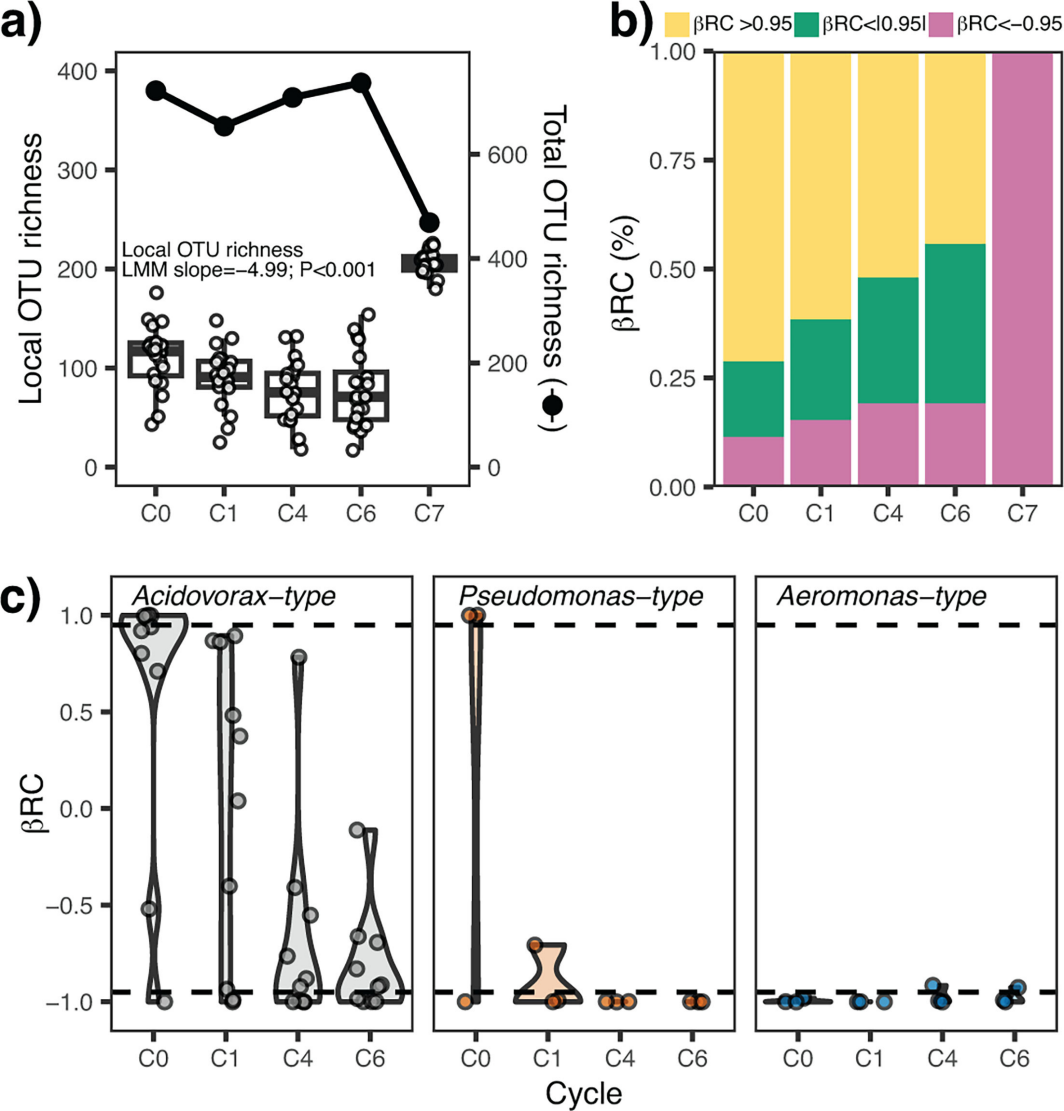
**(a**) OTU richness per microcosm (left axis) and total OTU richness across all the microcosms (right axis) over the cycles. (**b**) Percentage of Raup–Crick (βRC) pairwise dissimilarities among the 20 microcosms per cycle according to whether they are less (βRC > 0.95)/more (βRC < −0.95) similar than expected by chance, or they do not differ from stochastic assembly processes βRC < |0.95|. (**c**) Pairwise βRC index computed for microcosms belonging to the same community type over the growth cycles. Dashed lines represent βRC values of 0.95 and −0.95.

Local operational taxonomic unit (OTU) richness per microcosm significantly decreased throughout the cultivation cycles, from 111 ± 33 OTUs at C0 to 78 ± 38 OTUs at C6 (linear mixed model, *P* < 0.001). By contrast, the total number of OTUs in all microcosms tended to remain stable from C0 to C6 (CV = 5.1%, Fig. 2a). Most C0 communities were more dissimilar than expected by chance (β_RC_ > 0.95). The proportion of community pairs more dissimilar than expected by chance gradually decreased over the cycles (from 73% to 46% of the pairwise comparisons). By contrast, pairs more similar than by chance (β_RC_ < −0.95) increased, from 11% at C0 to 19% at C6 (Fig. 2b).

### Community type affects changes in composition and bulk parameters

We categorized communities according to dominant taxon. *Acidovorax*-type communities transitioned from predominantly dissimilar pairs at C0 (β_RC_ > 0.95) to increasingly more similar in subsequent cycles (Fig. 2c). An even steeper decrease of β_RC_ was observed for *Pseudomonas*-type communities, with all pairwise comparisons being <−0.95 by growth cycle 4 (C4). *Aeromonas*-type communities were already highly similar (β_RC_ < −0.95) at C0 and remained stable over the growth cycles (Fig. 2c).

**Fig 3:**
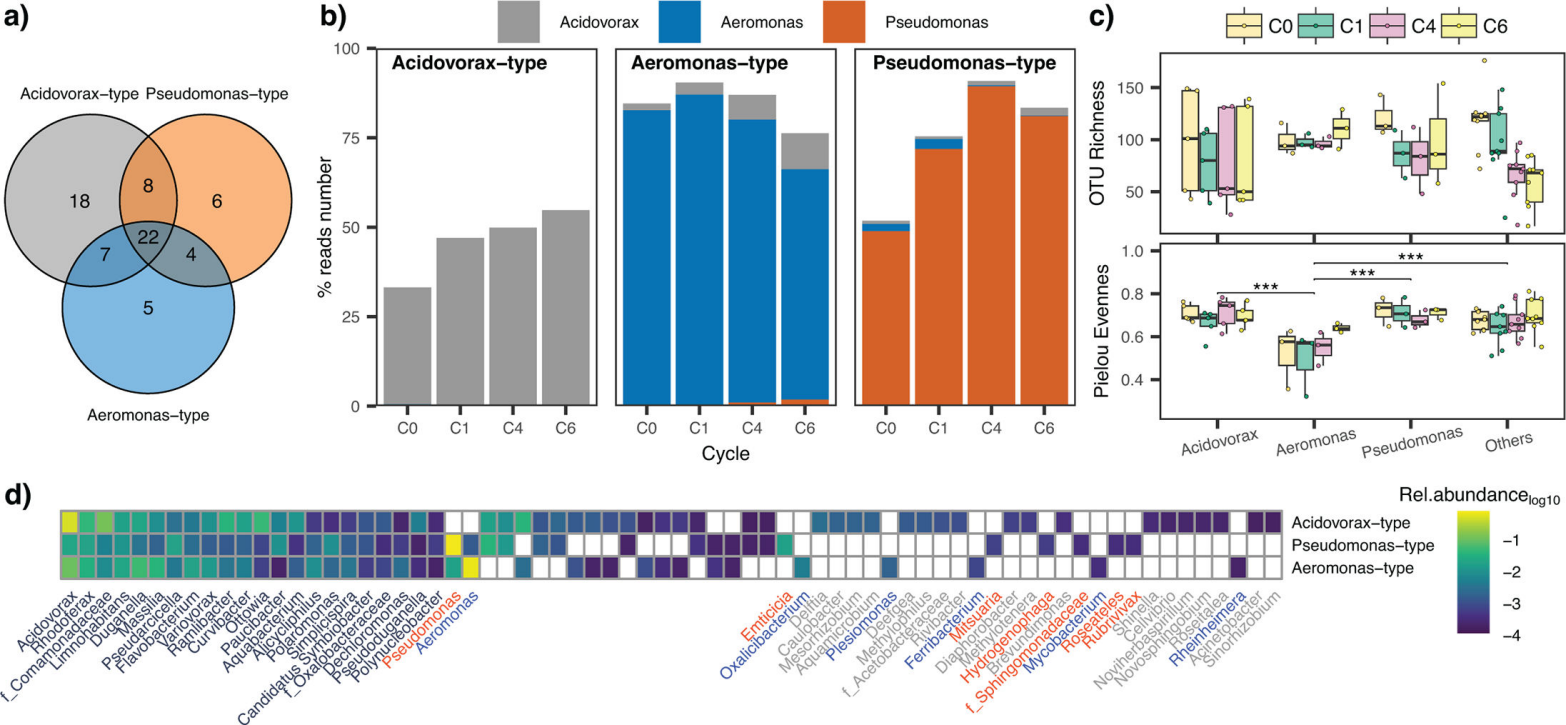
**(a**) Specific and shared genera in community types, i.e., communities dominated by *Acidovorax* (*n* = 5), *Pseudomonas* (*n* = 3), or *Aeromonas* (*n* = 3) at C6. (**b**) Relative abundance of the three representative genera in their community type (% reads number). (**c**) OTU richness and Pielou evenness indices per community type in the experimental cycles. (**d**) Relative abundances of genera in the three community types.

The three community types together harbored 82% of all genera detected at C6 (Fig. 3a). The three dominant genera only co-occurred during the early growth cycles (Fig. 3b). The community types exhibited similar OTU richness (repeated measurement analysis of variance [ANOVA], *P* > 0.05), but *Aeromonas*-type communities were less even (Pielou’s evenness) than the other two types and the unique communities (repeated measurement ANOVA, *P* < 0.001) (Fig. 3c). Twice as many genera were exclusive to the *Acidovorax*-type communities than to those of the other two types (Fig. 3d).

**Fig 4:**
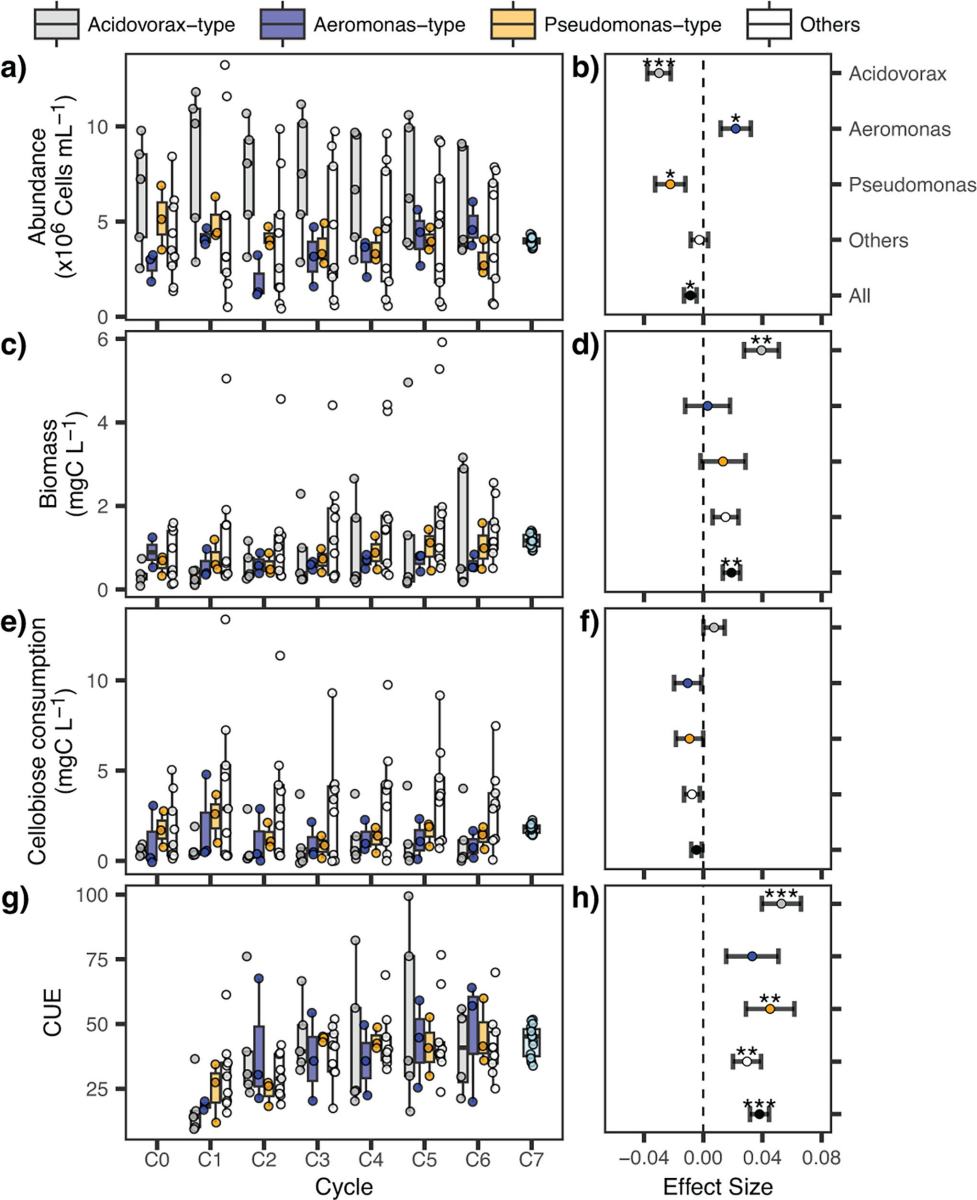
Left panels: (a) cell abundances, (**c**) biomass, (e) cellobiose consumption, and (g) CUE over the six cycles of semi-continuous growth (C1 to C6) and the homogenizing dispersal event (C7) in the three microcosms community types and the set of other assemblages. Right panels: slopes derived from the mixed linear model for (b) cell abundances, (**d**) biomass, (**f**) cellobiose consumption, and (h) CUE. The dashed lines indicate slope = 0. Asterisks: *, *P* < 0.05; **, *P* < 0.01; ***, *P* < 0.001.

Bacterial abundance per microcosm did not correlate with biomass (Fig. S2): bacterial abundance decreased over the growth cycles (Fig. 4a and b), whereas communities produced increasingly more biomass, i.e., larger cells (Fig. 4c and d). Cellobiose consumption in the microcosms did not significantly change over cycles (cycle, repeated measurement ANOVA, *P* = 0.126, Table S3; Fig. 4e and f), but carbon use efficiency (CUE) increased 1.5-fold. (Fig. 4g and h).

Community type affected both, magnitude and temporal changes of some community bulk parameters (Fig. 4). *Acidovorax*-type communities had higher bacterial abundances than *Pseudomonas* and *Aeromonas* types (Fig. 4a). Bacterial abundances decreased over the growth cycles in *Acidovorax*- and *Pseudomonas*-type communities but increased in the *Aeromonas*-type ones (Fig. 4b). The overall increase in biomass was mainly driven by the steep rise in the *Acidovorax*-type communities (Fig. 4c and d). By contrast, the rate of CUE increases only slightly varied between community types (Fig. 4g and h). Communities of unique composition showed high variability in bulk parameters amongst each other: some unique communities had more than three times higher biomasses and cellobiose consumption rates than the average of the common community types (Fig. 4c and e).

### Changes in genus-level competitiveness over time (Elo-rating)

We used Elo-rating (originally developed to rank chess players across multiple tournaments [21]) as an index to assess the overall performance of individual taxa within the metacommunity over time, i.e., how often they occurred in the microcosms, and their relative abundances in these communities.

**Fig 5:**
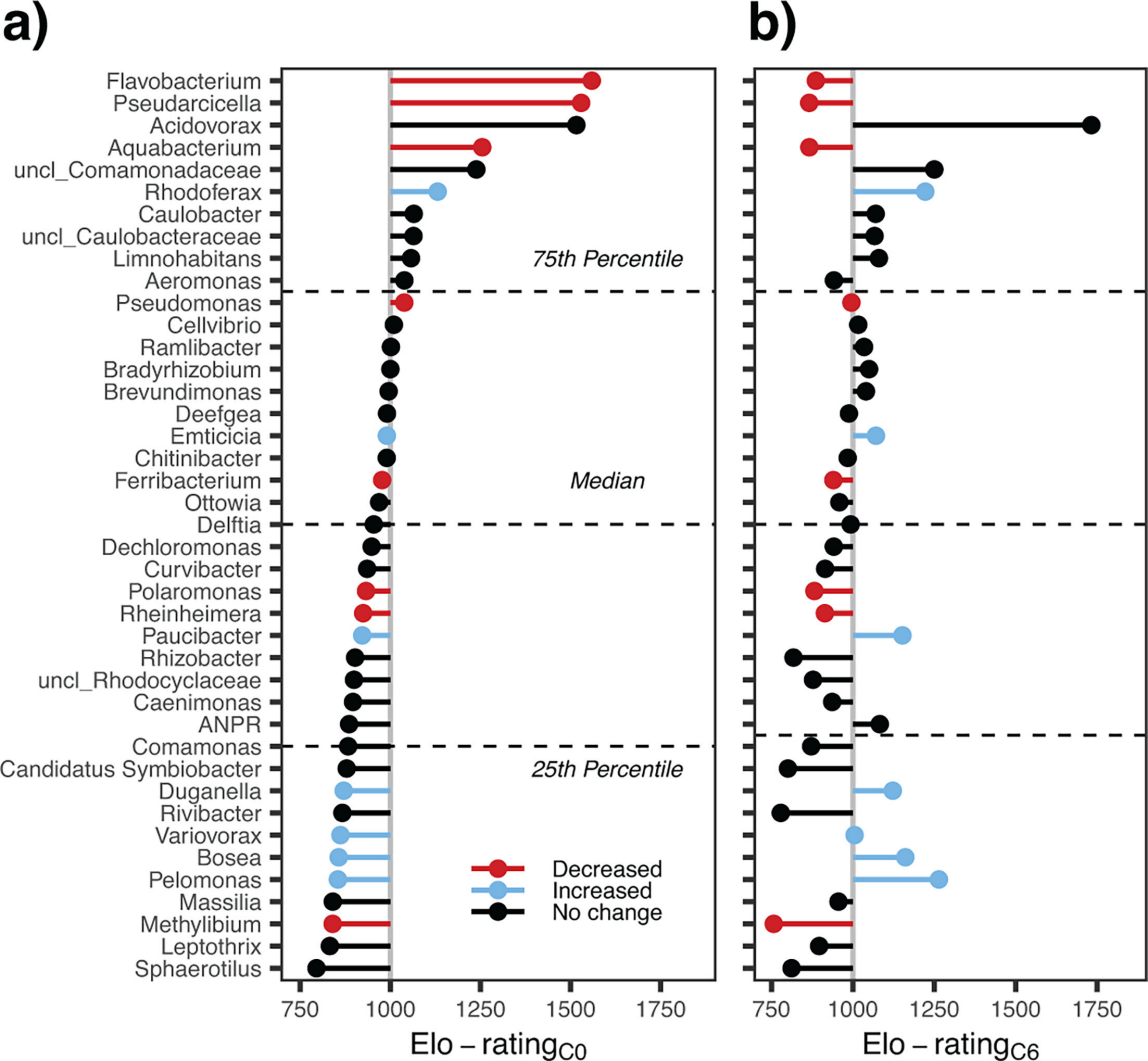
Elo-rating of genera after (a) C0 and (b) C6 contributing >0.1% to average read numbers. Colored dots indicate genera significantly increasing (blue) or decreasing (red) Elo-ratings over the experimental cycles (*n* = 4, Spearman rank correlation). Black dots present genera with no significant change in Elo-ratings.

The Elo-rating was used to generate rank distributions of genera with >0.1% relative abundances in at least one microcosm between C0 and C6 (Fig. 5). Several successful primary colonizers (rating above 75th percentile at C0), such as *Flavobacterium*, *Pseudarcicella*, and *Aquabacterium,* significantly declined in their Elo-rating, whereas *Rhodoferax* became more competitive (Fig. 5). While most of the initially less competitive genera did not significantly change or even decreased in Elo-rating (e.g., *Polaromonas*, *Rheinheimera,* and *Methylibium*), several others, such as *Paucibacter*, *Duganella*, *Variovorax*, *Bosea,* and *Pelomonas* significantly improved in competitive performance over the cycles (Spearman rank correlation, *P* < 0.05; Fig. 5).

The Elo-rating of *Acidovorax, Aeromonas,* and *Pseudomonas* were all above the median. While the ratings of the former two did not change over the cycles, *Pseudomonas* slightly but significantly decreased (from 1,039 to 996, Spearman rank correlation, *P* < 0.05; Fig. 5), reflecting its increasingly restricted occurrence across microcosms. *Acidovorax* had the third-highest Elo-rating in C0, and the highest one by a large margin in C6.

### Homogenizing dispersal event

**Fig 6:**
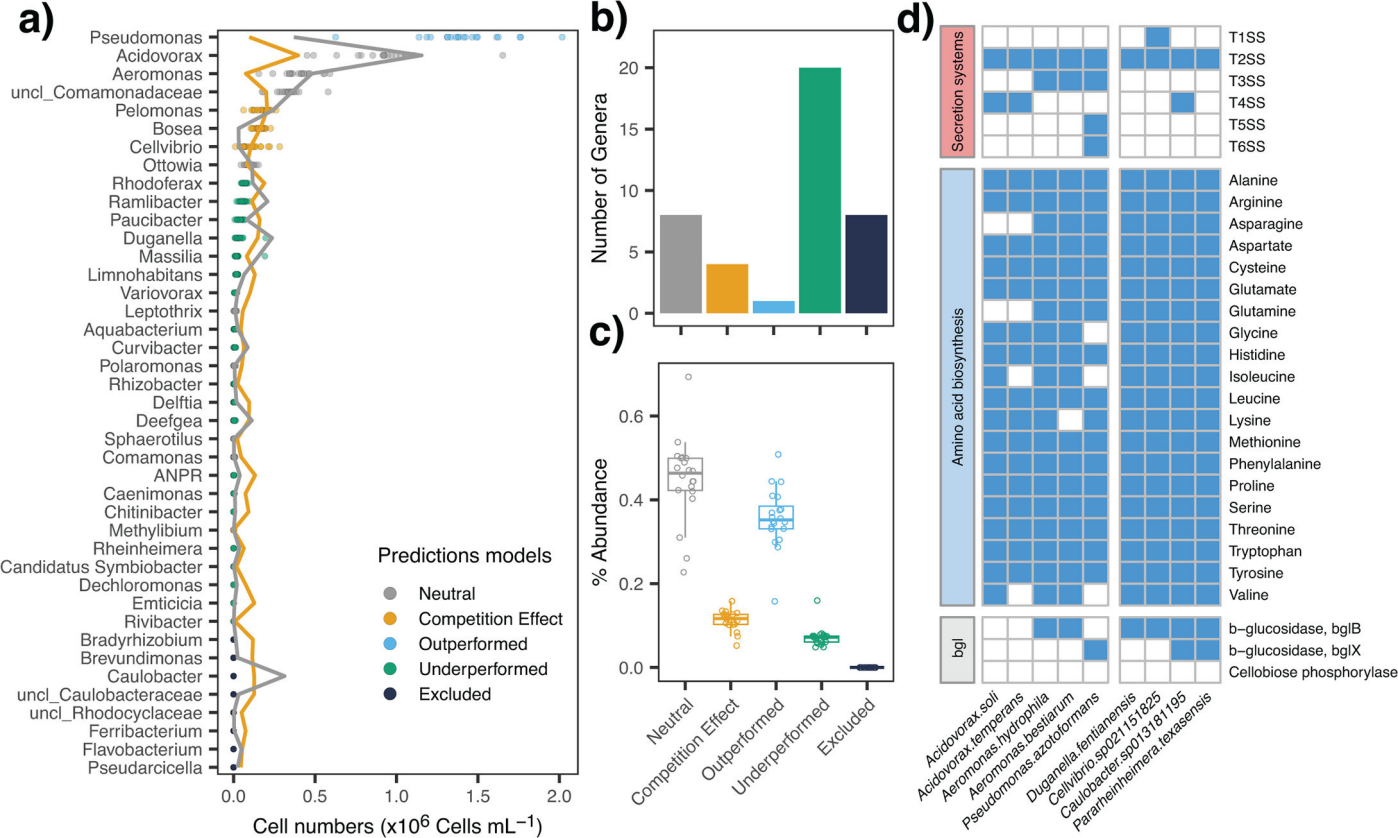
**(a**) Homogeneous dispersal event (Cycle 7) and per genus performance prediction. Relative abundance of genera predicted from their abundances in C6 with a neutral model (gray line) or from their competitiveness, i.e., Elo rating (orange line). The observed relative abundance of genera in each microcosm of C7 is depicted with symbols whose color indicates if the observed abundance matched a model (gray or orange), if the genera were overperforming (blue), underperforming (green), or were undetectable in C7 (black). (**b**) Number of genera and (c) corresponding read proportions predicted by the neutral, the competitiveness model, that out- or underperformed for both models and excluded ones from C7 communities. (**d**) Selected genomic traits in metagenome-assembled genomes (MAGs) of the dominant taxa in C7 (*Acidovorax soli*, *Acidovorax temperans*, *Aeromonas hydrophila*, *Aeromonas bestiarum,* and *Pseudomonas azotoformans*) and of taxa that dominated in single microcosms in C6 (*Duganella fentianensis*, *Cellvibrio sp02115825*, *Caulobacter sp013181195,* and *Pararheinheimera texasensis*).

We experimentally induced a homogenizing dispersal event (growth cycle 7, C7) using the 20 parallel microbial communities from C6 (Fig. 1a). Homogenizing dispersal produced highly similar microbial assemblages (β_RC_ < −0.95; Fig. 2b) dominated by the genera that already formed the highest abundances in 50% of the C6 microcosms (*Pseudomonas*, *Acidovorax*, and *Aeromonas,* Fig. 1b). Moreover, 20% of genera from C6 were not detected in C7, including some that represented a sizable fraction of the C7 inoculum (e.g., *Caulobacter*, Fig. 6a).

A neutral and a competitive model were compared in their power to predict the community composition after the homogenizing dispersal event (C7, Fig. 6a). The neutral model was based on the principle of mass effects (9, 22), i.e., the respective abundances of each genus in C6 microcosms explained their abundances in C7. It successfully predicted 20% of the genera, representing ~44.8% ± 9.9% of abundances (Fig. 6b and c). The competitiveness model, based on genus-specific Elo-ratings from C6, most accurately predicted the C7 abundances of three of the top 10 most abundant genera (*Pelomonas*, *Bosea,* and *Cellvibrio*), accounting for 11.2% ± 2.3% of abundances. Only *Pseudomonas* outperformed both predictors, whereas many rare genera performed worse than predicted by either model (Fig. 6c).

Bulk community parameters of the C7 communities were highly similar (Fig. 4). Biomass and CUE in C7 matched the average values of the C6 communities (Table S3), while total cell abundances and cellobiose consumption were ~13% and ~10% lower than before homogenizing dispersal (Table S3).

We processed the metagenomes from C6 and C7 to retrieve metagenome-assembled genomes (MAGs) that defined the C6 “community types,” which were affiliated with the most abundant taxa across the microcosms. From the dominant genera *Pseudomonas*, *Acidovorax,* and *Aeromonas*, we obtained 4, 15, and 4 MAGs from C6 and 4, 19, and 21 from C7, respectively (Table S4). Additionally, we retrieved nearly single MAGs from the most abundant taxa in microcosms of unique composition at C6, including the genera *Caulobacter, Cellvibrio, Dugannella,* and *Pararheinheimera* (Table S4).

Based on the ANI distance of the MAGs, a single *Pseudomonas* genotype, *P. azotoformans,* dominated in C7 communities (Table S5). *Acidovorax* and *Aeromonas* were each represented by two mutually exclusive genotypes, *A. temperans* or *A. soli*, and *A. hydrophila* or *A. bestiarum*, respectively (Tables S6 and S7).

### Genomic traits of dominant community members

We assessed genes for secretion systems, amino acid biosynthesis, and cellobiose degradation in the dominant genera at C7 and genera dominating at least one C6 microcosm (Fig. 6d). Type 2 secretion systems (T2SS) were found in all MAGs. Only MAGs affiliated with *Pseudomonas*, *Aeromonas, and Acidovorax* featured T3SS, T5SS, and T6SS, indicating strong competitive traits (23).

Of the studied taxa, only *A. hydrophila* was prototrophic for amino acids. *Pseudomonas* and *Acidovorax* MAGs each lacked synthesis pathways for specific amino acids (Fig. 6d). *Aeromonas* MAGs and *P. azotoformans* MAGs contained genes involved in cellobiose consumption (bgl-B and bgl-X, respectively), whereas *Acidovorax* MAGs did not. All MAGs dominating single microcosms at C6 were prototrophic for amino acids and featured genes coding for β-glucosidases (Fig. 6d).

## DISCUSSION

### Compositional and functional variability in the absence of dispersal

Dispersal limitation during initial colonization of the microcosms (Cycle 0) led to a set of stochastically assembled communities with high β-diversity (Fig. 1b) (8). Subsequent semi-continuous cultivation (i.e., zero dispersal rates) revealed contrasting effects of local isolation on different levels of diversity (24, 25) (Fig. 2a and b). Our findings align with observations in anaerobic bioreactors, where stochastically assembled communities decreased in richness during the transition to a deterministic regime (26). They also experimentally support microbial metacommunity models predicting that the absence of dispersal will strengthen local biotic selection (11). Other experimental systems, such as freshwater nematode metacommunities, maintained stable diversity levels despite prolonged local isolation (27). This difference in our findings is probably due to resource availability: our experimental system relied on a limited number of resources (cellobiose and glucose), thereby promoting biotic selection and diversity loss. By contrast, nematodes could exploit a wide range of resources, including bacteria, microphytobenthos, protists, meiofauna, or organic debris (28). This likely led to reduced substrate competition and niche separation, which in turn stabilized diversity during segregation.

Interestingly, total compartmentalization rarely resulted in the extinction of OTUs (or their decrease below the detection level of our method) at the metacommunity level, as illustrated by stable γ-diversity over the growth cycles (Fig. 2a). Instead, it led to a decline in OTU occurrence or even to “endemism” within single microcosms, illustrating both their redundant roles in most local assemblages and their likely dependence on positive biotic interactions to circumvent elimination (29). This gradual “purging” of OTUs from microcosm communities during their transition to more deterministic assembly processes was also the main driver of increasing β-diversity (Fig. 2b).

The simultaneous increase in biomass and CUE of microcosm communities during growth cycles contrasted with stable cellobiose consumption rates (Fig. 4). This suggests that their improved performance was not directly driven by specialized taxa that could degrade the primary resource, but is best attributed to increasing efficiency of using the “common good,” cellobiose-derived glucose. Other biotic interactions might have also contributed to reduced energy waste, e.g., enhanced cross-feeding on secreted metabolites (30), or energy reallocation from metabolically costly competitive traits to growth yield due to reduced interspecific competition (decrease in α-diversity; Fig. 2a) (31). Our findings also speak for MacArthur’s minimization principle in communities developing under competition at stable conditions: unutilized resources decreased with community maturity due to niche complementarity among species (32). While this concept has received little attention (33), an experimental study using synthetic phytoplankton communities confirmed its predictions regarding biomass production (34). Our results extend these findings by showing that cellobiose-derived carbon was increasingly fixed into microbial biomass across growth cycles, irrespective of community structure (Fig. 4g and h). Finally, microevolutionary adaptation toward more efficient glucose consumption (35) could also explain the increasing efficiency.

Originally designed for assessing dyadic interactions within game tournaments (21), Elo-rating has been used in biology to assess the social structure in primates (36). Our implementation demonstrated suitability for metacommunity analysis by clearly highlighting *Acidovorax* as the overall “winner” across multiple communities (Fig. 1b). More importantly, it gave insight into subtle community re-arrangements during growth cycles that would have been challenging to detect without context-dependent measure, e.g., the increasing importance of *Paucibacter*, *Bosea*, or *Pelomonas*, and the concomitant decline of *Flavobacterium*, *Pseudodarcicella*, or *Aquabacterium* (Fig. 2 and 5). Additionally, it was the best predictor for the performance of three of the top ten most abundant genera after metacommunity mixing (Fig. 6). Thus, Elo-rating could be an additional tool to assess the overall success of taxa in metacommunities based on their competitive performance within and among local assemblages.

### Community types

Stochastic assembly processes can generate compositional and functionally distinct communities (7). We show that dispersal limitation within metacommunities may produce recurrent community types with different carrying capacities (Fig. 4a and d), evenness (Fig. 3c), and subsets of exclusively associated taxa (Fig. 3d). We defined types from genera dominating three or more microcosms (*Acidovorax*, *Pseudomonas,* and *Aeromonas*) (Fig. 1b and 3). These genera are typically members of the rare aquatic biosphere that proliferate upon input of organic matter or in substrate-rich microniches (37, 38). *Acidovorax* was initially seeded into all microcosms, and all local populations survived over the growth cycles. Since these bacteria lack a known cellobiose degradation mechanism (Fig. 6d), cellobiose-derived glucose must have been available to them as a “common good.” By contrast, both *Pseudomonas* and *Aeromonas* were dispersal-limited and more vulnerable to biotic selection. In general, the community types self-stabilized: while initial stochastic dispersal established the state for subsequent development (Fig. 1B, C0), the biological interaction cycles resulted in their deterministic “purification.” This led to stable or increasing within-type similarity against a background of increasing metacommunity-level β-diversity (Fig. 2a and c).

### Effects of homogenizing dispersal on the metacommunity

Experimental homogenizing dispersal increased α-diversity and similarity (lower β-diversity) of local microcosm communities but led to a reduction of total metacommunity (γ) diversity (Fig. 2a and b). These observations do not align with the theoretical predictions for a fully connected metacommunity subjected to high dispersal rates (9, 39). Thus, the effects of a singular coalescence event differ from the source-sink dynamics resulting from a continuous process of connectivity. Moderate local species sorting in post-coalescence microcosms was suggested by the large proportion of abundances of individual genera explained by the parent communities (i.e., by the neutral model, Fig. 6c). The appearance of novel positive interactions among previously allopatric populations may also have contributed to the increased local diversity (40). Since our experiment was limited to a single growth cycle after coalescence, we cannot assess if this high initial diversity was only temporary. A gradual loss of diversity over a 6-week period was demonstrated in an experimental study of mixed soil and carcass communities (41).

Upon coalescence, the heterogeneous assemblages transitioned to novel, more uniform communities that differed from all source communities (Fig. 1b). Homogenization of synthetic bacterial communities has been observed already at low dispersal rates (42). Comparable findings have been reported from long-term field observations at the landscape scale: the anthropogenic connection of freshwater bodies (related to the construction of a reservoir) led to the homogenization of the zooplankton metacommunity (43).

Coalescence also led to functional uniformity (Fig. 4): community performance didn’t improve after mixing but instead stabilized around the median value of the parent communities (Table S3). This contrasts with previous observations where the best-performing parent community dictated both, the structure and function of post-coalescence methanogenic assemblages (44). The loss of functional variability most likely resulted from the disproportional decline or extinction of functionally distinct taxa (Fig. 6d) that dominated in single C6 communities and significantly contributed to this variability (Fig. 4c and e). These “endemic” populations, *Rheinheimera*, *Duganella,* and *Caulobacter* (Fig. 1b), proved to be extremely vulnerable to competitive exclusion (45–47). Thus, our experimental observations shed light on how homogenizing dispersal can affect species trait distributions and lead to a loss of functional variability at the metacommunity level (Fig. 4), thereby potentially altering ecosystem functioning through the replacement of specialists at the expense of generalists (48) and functionally inefficient species (49).

The post-coalescence dominance of one genotype of *Pseudomonas* from a single isolated microcosm, *P. azotoformans,* conspicuously exceeded our predictions (Fig. 1 and 6; Table S5). The analysis of the corresponding MAGs revealed that *P. azotoformans* was the only abundant community member that featured T5SS and T6SS. These secretion systems confer competitive advantages to pseudomonads by delivering effectors such as nucleases, amidases, hydrolases, or phospholipases to neighboring bacterial cells and the external milieu (50, 51).

Taken together, our findings suggest that dispersal limitation may play a key role in defining community performance, by stochastically segregating highly efficient “bottom-up” specialists from taxa that outcompete them via negative biotic interactions (52, 53). This holds relevance for a rational selection of stable microbial assemblages for both industrial and ecosystem restoration purposes (54). Specifically, we demonstrate the feasibility of a “top-down” design approach to optimize degradation efficiency in synthetic communities by producing rare variants that outperform the more common types: the highest levels of cellobiose degradation occurred in a unique dominated community stable over the six growth cycles (i.e., *Caulobacter*; Fig. 1b) but did not survive community coalescence (Fig. 6a). Our findings thus provide a potential alternative to classical bottom-up approaches (55), by allowing for intrinsic biotic relationships from initial stochastic assembly to serve as stabilizing force during deterministic selection (56).

## MATERIALS AND METHODS

### Sampling site and experimental design

Water for the inoculum of the experiment was collected at 5 m depth from the prealpine oligo-mesotrophic Lake Zurich (Switzerland) on 4 October 2019. It was prefiltered using 0.8 µm pore size filters (polycarbonate membrane, Whatman, Maidstone, UK) via a peristaltic pump (Ismatec, Wertheim, Germany) to exclude potential grazers and other eukaryotes from the microcosms.

The experiment comprised three phases: initial colonization of sterile microcosm environments, six growth cycles in semi-continuous culture, and a final “coalescence event” (Fig. 1a). Bacterial communities were grown in artificial lake water (ALW) (8) supplemented with glucose (10 µmol L^−1^) and its dimer, cellobiose (100 µmol L^−1^). This setup aimed to mimic the natural pool of dissolved organic carbon in aquatic systems, i.e., low concentrations of labile and higher concentrations of recalcitrant compounds (57). Microcosms consisted of 200 mL Erlenmeyer flasks incubated at 20°C in dark conditions. At the end of each growth cycle, samples for substrate utilization, bacterial growth, and biomass were collected.

For the initial colonization phase (growth cycle 0, hereafter C0), the filtered lake water was inoculated into ALW (1:100), homogenized, and distributed over 20 microcosms. This procedure promotes the dispersal limitation of rare lake bacteria that thrive in the provided environmental conditions (8). Microcosms were incubated for 6 days until bacteria reached the stationary phase. For the semi-continuous cultivation phase, 20 mL from each microcosm was transferred into 180 mL of substrate-supplemented ALW in a new microcosm. These cultures were incubated for 4 days between subsequent transfers, for altogether six growth cycles (C1 to C6; Fig. 1a). In the final phase, 20 mL from each of the 20 communities were mixed to simulate homogenizing dispersal, diluted with ALW (1:10), homogenized, and distributed across 20 microcosms. These microcosms were incubated for 4 days (C7; Fig. 1a).

### Bacterial abundances and biomass

For bacterial enumeration, 1 mL portions were fixed with formaldehyde (2% final concentration), stored at 4°C, and measured within 24 h. Fixed samples were stained with SYBR Green and analyzed on a CytoFLEX flow cytometer (Beckman Coulter, Indianapolis, IN, USA). For biomass determination, 50 mL aliquots were filtered onto precombusted 0.22 µm pore size GF/F filters (Tisch Scientific, 450°C for 6 h) and stored in small aluminum containers at −20°C until analysis. The total organic carbon was quantified on a dry combustion module cavity ring-down spectrometer (Picarro Inc, Santa Clara, CA, USA). Filters were combusted at 950°C, and the resulting CO_2_ was quantified. Standards with a known C-content (*Miscanthus*) served as the reference for calibration.

### Substrate quantification

Glucose and cellobiose concentrations were determined by high-performance liquid chromatography (1260 Infinity series, Agilent Technologies, Santa Clara, CA, USA) coupled with mass spectrometry (API 5000 triple quadrupole, AB Sciex, Baden, Switzerland; HPLC‒MS). Aliquots (1.5 mL) were filtered through 0.1 µm membrane filters (Polyethersulfone, Infochroma AG, Goldau, Switzerland) and stored at −20°C until analysis. Measurements were conducted as described (58), using sucralose (2 µmol L^−1^) as the internal standard. Data were acquired using Analyst v1.6.1 software (AB Sciex), and chromatograms were analyzed via MultiQuant v2.1 (AB Sciex).

### Carbon use efficiency

CUE was calculated as the ratio between the biomass produced and the corresponding amount of carbon (combined concentrations of glucose and cellobiose) consumed during each cycle.

### DNA extraction

At the end of cycles C0, C1, C4, C6, and C7, 100 mL from each microcosm were filtered onto a 0.22 µm pore size filter (GPWP, Millipore, Darmstadt, Germany), stored at −20°C until DNA extraction with the DNeasy PowerBiofilm Kit (Qiagen, Germany). Metagenomic DNA was sequenced using the Illumina shotgun NovaSeq 6000 platform (2 × 150 pb, NOVOGENE, Cambridge, United Kingdom).

### 16S rRNA genotyping

For bacterial community structure analysis, we retrieved reads from the C0, C1, C4, C6, and C7 metagenome sequences mapped to the 16S rRNA gene using published pipelines (59, 60). Forward and reverse reads were merged using BBmerge v38.86 (61) at default settings and filtered by length (>200 bp) using BBduck v38.86 (61). These pre-processed reads were queried against the SILVA SSU database using MMseqs2 (e-value 1e^−3^) (62) to identify RNA-like sequences. Bona fide 16S rRNA sequences were further compared by blastn (e-value 1e^−5^) with SSU-ALIGN v0.1 (http://eddylab.org/software/ssu-align/) against the SILVA 99NR database v138.1 (63). OTUs were constructed by BLAST (v2.9.0) analysis of the identified 16S rRNA sequences against SILVA that simultaneously had identity values >97% and alignment lengths ≥80% (64). Reads were rarefied to the read count of the lowest sample (2,911; Table S1).

### Genome assembly and functional annotation

Raw Illumina reads were quality and adapter trimmed using BBduck v38.86 (61) (qtrim=rl trimq=30). Reads were assembled per sample using MEGAHIT v1.2.9 (defaults settings, k-mer 29, 39, 49, 59, 69, 79, 89, 99). The metagenomic reads were mapped using BBmap v38.86 (61) against the assembled contigs. The abundance profile of assembled contigs was used for binning with MetaBAT2 (65). Completeness and contamination were assessed by CheckM v1.2.2 (66). Bins with contamination <5% were considered MAGs for further analysis (Table S4). MAGs were taxonomically classified with GTDB-tk v1.4.0 software (67) against the GTBD database release R07-RS207. Coding sequences were predicted via Prokka v1.12 (68) and annotated using the Kyoto Encyclopedia of Genes and Genomes (KEGG) (69). Metabolic reconstruction and genomic traits analysis were conducted with the KEGG mapping tools (https://www.genome.jp/kegg/mapper/reconstruct.html) using the previously annotated KO numbers. Proteins involved in cellobiose degradation (beta-glucosidases and cellobiose phosphorylases) we identified using the UniProtKB/Swiss-Prot protein database (release 2024_02 v1).

### Genomic traits associated with biological interactions

We searched for genomic traits associated with positive and negative biological interactions. Amino acid biosynthesis pathways were analyzed to detect auxotrophic taxa. Auxotrophies for essential metabolites increase metabolic interdependencies within microbial communities, thereby promoting positive interactions (70). Genes associated with bacterial secretion systems were assessed as proxies for competitive advantages (23). Since some secretion systems can be associated with positive interaction (i.e., cell-cell communication), we operationally classified them into weak or strong competitive traits. Weak competitive traits comprised type I, II, and IV secretion systems (T1SS, T2SS, T4SS) that relate to host-pathogen interactions, and participate in bacterial genetic exchange (23). In contrast, strong competitive traits encompassed type III, V, and VI secretion systems (T3SS, T5SS, T6SS), which can provide a direct competitive advantage by enhancing survival and invasion capacity through the release of toxins and effectors into the environment or neighboring cells (71).

### Assessment of competitiveness and predictions for the homogenizing dispersal event

Competition among genera in our low-complexity communities at C0, C1, C4, and C6 was assessed by a multiplayer version of the Elo-rating index used to compare the performance of players across multiple matches in gaming (72). For calculations, we used the multielo-package v0.4.0 implemented in Python (https://github.com/djcunningham0/multielo). Elo-ratings rely on the accuracy of a scoring function, for which we fitted an exponential decay function to the distribution of bacterial read numbers from cycle C0 to C6 (*R*^2^ = 0.90, *P* < 0.001; Fig. S1a). Each genus obtained a score (So_*G*_) based on its ranking in the community. Subsequently, the Elo rating per genus (Elo_*G*_) in each microcosm was calculated as


(1) $${Elo}_{G,n}={Elo}_{G,n-1}+K\left(N-1\right)\left({So}_{G}-{Se}_{G}\right),$$

where *K* (default = 32) corresponds to the sum of points per microcosm after all pairwise “matches,” *N* is the number of bacterial genera, and Se_*G*_ is the per genus expected score if all community members have the same winning probability. Elo-rating was calculated per cycle and sequentially updated through the 20 microcosms. Because the order of the microcosms can influence Elo-rating results, we randomized the order of microcosms (*n* = 1,000), and the average Elo-rating was reported.

The effects of the homogenizing dispersal event at the genus level were assessed by comparing whether the final proportions of genera in C7 were better predicted by competitiveness (their Elo-ratings in C6) or neutral processes (their respective abundances in the inoculum for C7). To calculate the competitive scenario, we first scaled the Elo-ratings in C6 by subtracting the minimum rating:


(2) $${Elo}_{G^{\prime }}={Elo}_{G}-\min\left({Elo}_{G}\right)$$

The scaled Elo-rating was normalized from 0 to 1 for further comparison with the observed abundances:


(3) $${Elo.norm}_{G^{\prime }}=\frac{{Elo}_{G^{\prime }}}{\sum \left({Elo}_{G^{\prime }}\right)}\times median\left(Cell\;counts_{C6}\right),$$

where 𝐸𝑙𝑜.𝑛𝑜𝑟𝑚_*G*_′ represents the expected abundance of the individual genus after the homogenizing dispersal event according to their competitiveness.

The expected abundance of the individual genus according to the neutral scenario was estimated by multiplying the relative read number per genus (*G*) with cell abundances per microcosm (*i*) from the C6. These results were summed up across microcosms (*n*), as follows:


(4) $${Abundance.neutral}_{G}=\sum_{i}^{n}{(relative\;read\;numbers}_{G,i}\;\times {cell\;count}_{G,i})$$

Abundance.neutral_*G*_ was normalized by the sum of cell counts across microcosms (*n* = 20).

### Statistical analysis

Statistical analyses were conducted using R (73). The modified Raup–Crick index (β_RC_) was used to assess the importance of community assembly processes using the Bray‒Curtis distance (74) at the OTU level with the R package NST (75). The β_RC_ performs a pairwise evaluation of community turnover based on a null model in which taxa are randomly shuffled among all communities. It indicates whether community pairs are more (β_RC_ < −0.95) or less (β_RC_ > 0.95) similar than expected by chance, or if turnover does not differ from the stochastic assembly (|β_RC_| < 0.95). Community pairs more similar than by chance are expected to be influenced by either homogenizing dispersal or homogeneous selection, while community pairs less similar than by chance are influenced by dispersal limitation.

Distinct community types were defined by genera that were both, abundant and prevalent (76), i.e., that had the highest read proportions of all genera at C6 in at least three microcosms. The average community composition of these types was derived from C6 microcosms. One-way repeated-measurement ANOVAs were performed to evaluate if community type affected alpha diversity (Richness, Pielou’s evenness) and bulk properties (bacterial abundance, biomass, and CUE). Normality and homoscedasticity were tested by Kolmogorov‒Smirnov and Levene tests, respectively. Linear mixed models were fitted to the bulk property values of community types from C0 to C6 to assess potential in or decrease during the semi-continuous cultivation phase (“lme” function, R package nlm). Spearman rank correlations of Elo-rating scores versus cycle number were used to test if the competitive performance of individual genera significantly changed between C0 and C6.

One-sample Wilcoxon or one-sample t-tests (depending on data distribution) were performed to assess if the abundances of individual genera after the homogenizing dispersal event in the C7 microcosms (*n* = 20) were more accurately predicted by the neutral or the competitive model (or by neither). The abundances predicted by either model were considered null hypotheses (*h*_0_). If neither predictor deviated from *h*_0_, the model yielding the higher *P*-value was selected. Genera with significantly higher or lower abundances than predicted by both models were classified as over- or underperforming, respectively. Multiple testing was adjusted for by the Benjamini-Hochberg method.

## Data Availability

R-scripts used for the data analyses have been published on GitHub (https://github.com/angelrainf/cycles.dispersal). Sequence data and metagenome-assembled genomes used in this study are deposited in the European Nucleotide Archive at EMBL-EBI (accession number PRJEB73309).

## SUPPLEMENTAL MATERIAL

Supplemental Figures msystems.01403-24-s0001.docx Figures S1 and S2.

Supplemental Tables msystems.01403-24-s0002.xlsx Tables S1 to S7.

ASM does not own the copyrights to Supplemental Material that may be linked to, or accessed through, an article. The authors have granted ASM a non-exclusive, world-wide license to publish the Supplemental Material files. Please contact the corresponding author directly for reuse.
