# Supplementary material for: Dispersal shapes compositional and functional diversity in aquatic microbial communities
Source: mSystems. 2024 Nov 18;9(12):e01403-24. doi: 10.1128/msystems.01403-24 (PMC11651098; doi:10.1128/msystems.01403-24)
Supplement: Supplemental Figures — Figures S1 and S2. [file msystems.01403-24-s0001.docx]

**Supplementary Information for**

**Dispersal shapes compositional and functional diversity in aquatic microbial communities**

**Authors:** Angel Rain-Franco^1*^(angel.rain@limnol.uzh.ch), Alizée Le Moigne^1,2^ (alizee.lemoigne@limnol.uzh.ch), Lucas Serra Moncadas^1^ (lserra@limnol.uzh.ch), Marisa O. D. Silva^3^ (marisa.silva@oncyt.com), Adrian-Stefan Andrei^1^ (stefan.andrei@limnol.uzh.ch) & Jakob Pernthaler^1^ (pernthaler@limnol.uzh.ch)

**Affiliations:** (1) Limnological Station, University of Zurich, Zurich, Switzerland; (2) Institut National de la Recherche Scientifique (INRS), centre Eau, Terre et Environnement, Québec, Canada; (3) onCyt Microbiology AG, Zurich, Switzerland.

^*^Corresponding author email: angel.rain@limnol.uzh.ch

Supplementary material include:

Supplementary Figure S1 to S2

Supplementary Table S1 to S7

**
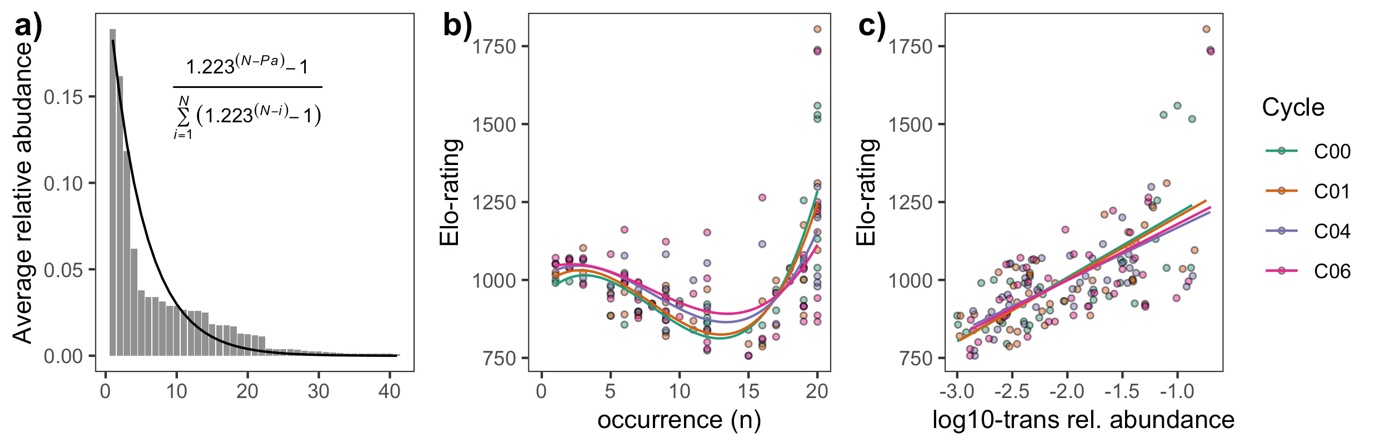
**

**Figure S1.** a) Exponential scoring function adjusted to the mean distribution of the genera that contributed >0.1% of the average relative number of reads from C0 to C6. The black line represents the adjusted scoring function (R^2^=0.90, P<0.001). b) Per genus Elo-rating vs the occurrence per microcosm. Lines represent the fitted polynomial curve (y = ax^3^ + bx^2^ + cx + d; C0: R^2^= 0.64, P<0.001; C01: R^2^= 0.53, P<0.001; C4: R^2^= 0.27, P= 0.002; C6: R^2^= 0.10, P= 0.070). c) Per genus Elo-rating vs their respective log10 transformed average relative abundance.

**
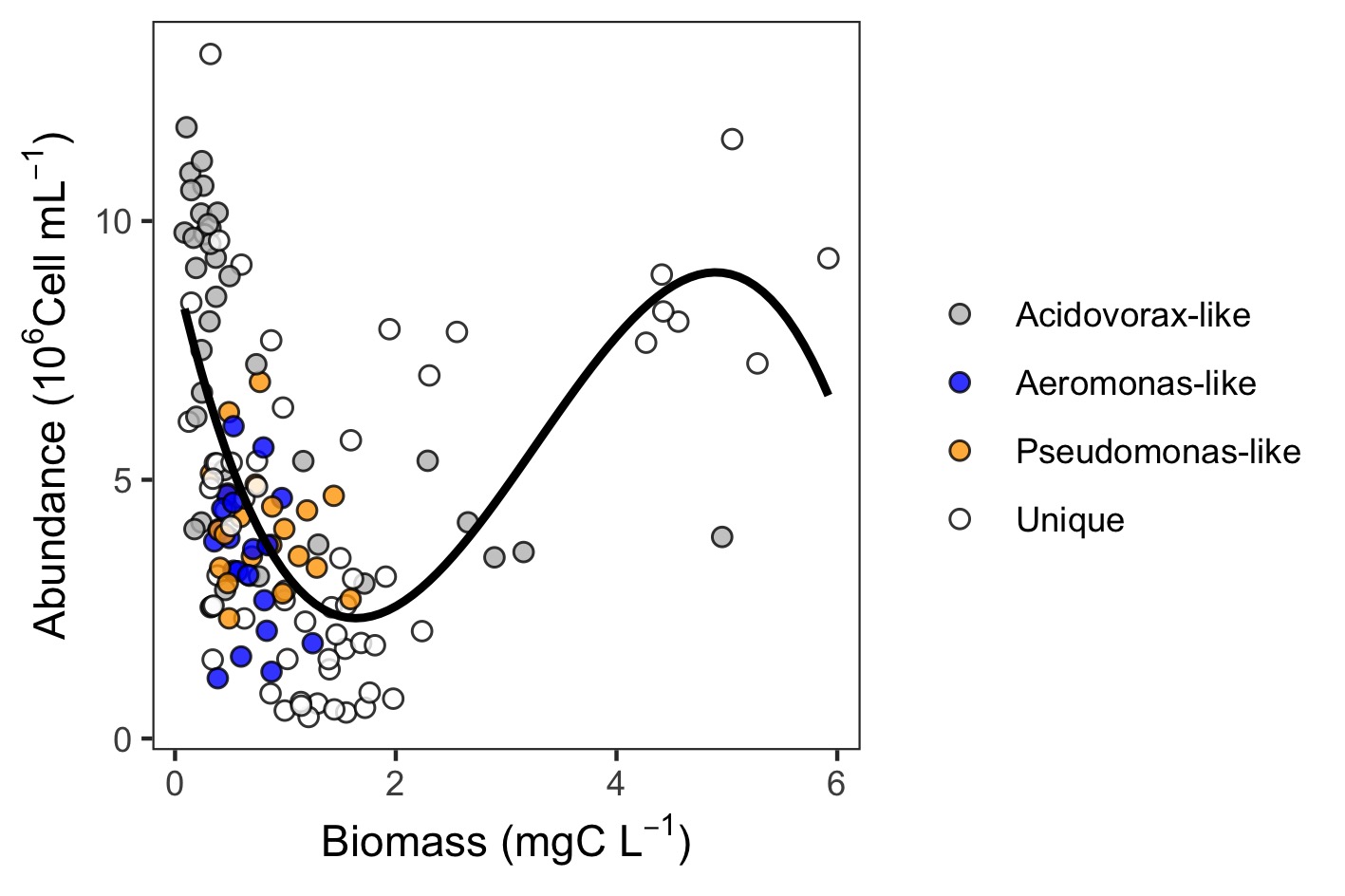
**

**Figure S2.** Relationship between the community abundance and biomass per microcosm from the dispersal limitation event and the cycles of biological interactions (C0 to C6). Line represents the fitted polynomial curve (y = ax^3^ + bx^2^ + cx + d; R^2^= 0.34, P<0.001).
